# Effect of Flax By-Products on the Mechanical and Cracking Behaviors of Expansive Soil

**DOI:** 10.3390/ma17225659

**Published:** 2024-11-20

**Authors:** Georgy Lazorenko, Anton Kasprzhitskii, Vasilii Mischinenko, Alexandr Fedotov, Ekaterina Kravchenko

**Affiliations:** 1 Climate Center, Novosibirsk State University, Pirogov Street, 2, Novosibirsk 630090, Russia; glazorenko@yandex.ru (G.L.); akasprzhitsky@yandex.ru (A.K.); m.vasbor@bk.ru (V.M.); fedotov5355@yandex.ru (A.F.); 2 Technological Faculty, Platov South-Russian State Polytechnic University, Prosveshcheniya St., 132, Novocherkassk 346428, Russia; 3 Academy of Biology and Biotechnology, Southern Federal University, Rostov-on-Don 344006, Russia

**Keywords:** soil improvement, expansive clay, natural fiber, flax tows, unconfined compressive strength, shear strength

## Abstract

Expansive soils, prone to significant volume changes with moisture fluctuations, challenge engineering infrastructure due to their swelling and shrinking. Traditional stabilization methods, including mechanical and chemical treatments, often have high material and environmental costs. This study explores fibrous by-products of flax processing, a sustainable alternative, for reinforcing expansive clay soil. Derived from the Linum usitatissimum plant, flax fibers offer favorable mechanical properties and environmental benefits. The research evaluates the impact of flax tow (FT) reinforcement on enhancing soil strength and reducing cracking. The results reveal that incorporating up to 0.6% randomly distributed FTs, consisting of technical flax fibers and shives, significantly improves soil properties. The unconfined compressive strength (UCS) increased by 29%, with 0.6% FT content, reaching 525 kPa, compared to unreinforced soil and further flax tow additions, which led to a decrease in UCS. This reduction is attributed to diminished soil–fiber interactions and increased fiber clustering. Additionally, flax tows effectively reduce soil cracking. The crack length density (CLD) decreased by 6% with 0.4% FTs, and higher concentrations led to increased cracking. The crack index factor (CIF) decreased by 71% with 0.4% flax tows but increased with higher FT concentrations. Flax tows enhance soil strength and reduce cracking while maintaining economic and environmental efficiency, offering a viable solution for stabilizing expansive clays in geotechnical applications.

## 1. Introduction

Expansive soils are highly plastic and typically composed of active clay minerals such as montmorillonite [1]. These soils exhibit significant swelling and shrinking behaviors, leading to the formation of numerous cracks during desiccation, wet–dry cycles, and freeze–thaw cycles [2,3]. The substantial volume changes in expansive soils due to variations in water content can cause extensive damage to the engineering infrastructure built upon them [1]. The associated economic losses are estimated at GBP £400 million [4], USD $1 billion [5], and USD $13 billion [6] per year in the UK, China, and the US, respectively. Because of these deformation characteristics, expansive soils require special attention. Given their widespread occurrence, it is crucial to ensure that construction on such soils is both stable and sustainable.

Traditional methods for improving expansive soils, such as mechanical and chemical stabilization, often involve materials and processes that may not be sustainable. Mechanical stabilization techniques, including deep soil mixing, cationic electrokinetic methods, and synthetic reinforcement, require significant material inputs [7]. Chemical treatments using lime, cement, and polymers are also common [3,8,9]. However, these methods can have a high carbon footprint, particularly cement stabilization [10], raising concerns about their environmental impact. For this reason, there has been a growing trend in recent years towards using natural fibers for soil reinforcement. For example, flax fibers, which are a by-product of flax production, can be used to improve the physical and mechanical properties of soil [11] instead of being disposed of in landfills or incinerated.

Flax (*Linum usitatissimum*) is a widely used natural fiber from the Linaceae family. Flax is one of the oldest and best-known bast fibers [12]. Flax fibers contain roughly 70% cellulose, 18% hemicellulose, and 5–10% lignin and wax [13]. The lignin content causes roughness, reducing smoothness and elasticity and increasing brittleness [12]. It is primarily derived from the stems of the flax plant, where the fibers are found in bundles of 10–40, held together by pectin. Each bundle contains individual fibers, which are polyhedral in shape and bound by pectin, contributing to their strength [14]. The fiber structure consists of a primary wall and a secondary wall with three layers. The fibers have a hollow interior called a lumen, formed after the cytoplasm inside the fiber degrades over time. They have a density of 1.4–1.5 g/cm^3^, which is lower than glass fiber’s density (~2.5 g/cm^3^), making them suitable for lightweight composite materials. Flax fibers have notable mechanical properties, including a tensile strength of 345–1100 MPa, a Young’s modulus value of 27.6 GPa, and elongation at a break of 2.7–3.2% [14]. Due to low elasticity, flax fibers do not return to their original shape after crumpling but are known for their high tensile strength, especially when wet, making them ideal for mats, nets, or geotextiles. Flax fibers are highly hygroscopic but poor thermal insulators [15].

Fiber reinforcement has a significant impact on the mechanical behavior of soil [16], which is why fiber reinforcement technology has been widely discussed, especially in relation to expansive soils. Tong et al. [17] conducted a study using a combination of bamboo strips and flax fiber to reinforce clay and found significant improvements in its mechanical properties. Through a series of tensile and triaxial shear tests, they discovered that this reinforcement method notably increased the cohesion and internal friction angle of the clay. El Hajjar et al. [18] conducted an experimental study focusing on addressing the issue of cracking due to desiccation-induced shrinkage strains in fine clayey soil. They investigated the reinforcement of soil using vegetal flax fibers arranged in orderly patterns to enhance its properties. The study revealed an eight-fold decrease in the crack ratio, demonstrating the effectiveness of soil reinforcement in reducing the crack opening. Ma et al. [19] explored the reinforcement mechanism of flax-fiber-reinforced clay through triaxial tests, examining various fiber content ratios and confining pressures. The study found that the shear strength of flax-fiber-reinforced clay significantly increased compared to pure clay, primarily due to enhanced cohesion. The shear strength peaked at a flax fiber content of 0.8% but decreased with further increases in the fiber content. Ma et al. [19] explained this behavior by stating that the uneven distribution of fibers in the clay disrupts the integrity of the soil matrix and leads to a decrease in contact between fibers and clay particles.

Despite these promising findings, there is still limited research on the specific effects of flax fiber reinforcement on expansive soils, particularly concerning strength improvement and crack reduction. This study aims to address this gap by systematically investigating the impact of flax fiber reinforcement on the strength and cracking characteristics of expansive soils. Specifically, the objective is to evaluate the effectiveness of using flax fiber by-products, which are often underutilized, as a sustainable and cost-effective alternative for soil stabilization. From this perspective, short, tangled, and non-aligned fibers known as flax tows (FTs) are of significant interest. These fibers are a by-product of the production of long flax fibers used in textiles. FTs are characterized by their high level of tangling and the presence of impurities such as flax shaves. Despite these drawbacks, flax tows have a market value of roughly half that of scutched flax [20], which makes them a more cost-effective reinforcement option. By analyzing how these by-products influence the mechanical properties and crack mitigation of expansive soils, this research seeks to provide insights into a more environmentally friendly approach to soil reinforcement.

## 2. Materials and Methods

### 2.1. Expansive Clay

**Figure 1:**
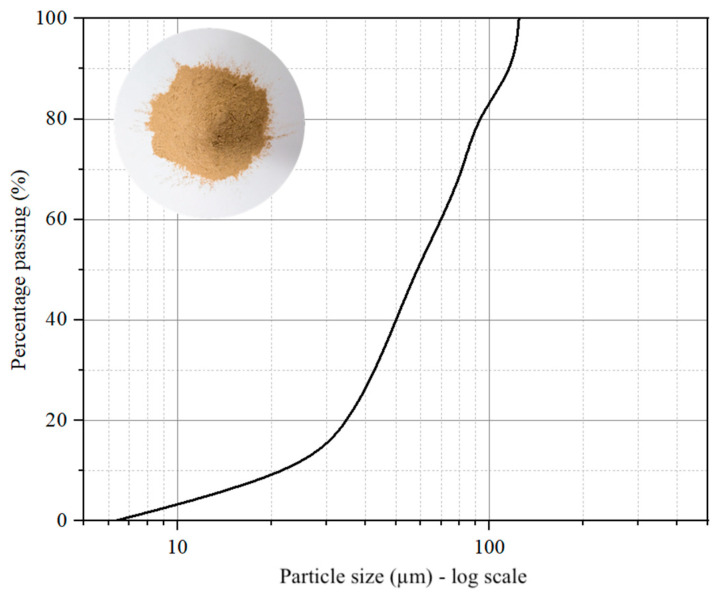
Grain size distribution curve of the selected soil.

In this study, montmorillonite clay was used, which was sourced from 30 km west of the regional center of Kurgan (Keto district of the Ural Federal District, Russia). The mineralogical composition of the studied soil was obtained through semi-quantitative XRD analysis using an Ultima IV multifunctional X-ray diffractometer (Rigaku Corporation, Tokyo, Japan). Chemical characterization of the samples was carried out using micro-XRF spectrometer M4 TRONADO (Bruker, Bremen, Germany). The grain particle size of the soil is shown in Figure 1. Also, it exhibited a low liquid limit (i.e., 32.3%) and is classified as CH (fat clay) in accordance with the Unified Soil Classification System (USCS). The liquid limit, plastic limit, plasticity index, optimum moisture content, maximum dry density, and other properties of the tested soil are reported in Table 1.

### 2.2. Flax By-Products

**Figure 2:**
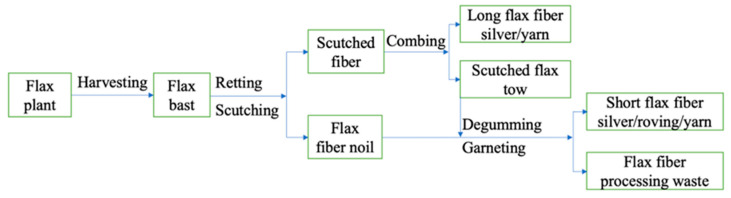
Preparation and application of scutched flax fiber [31].

**Figure 3:**
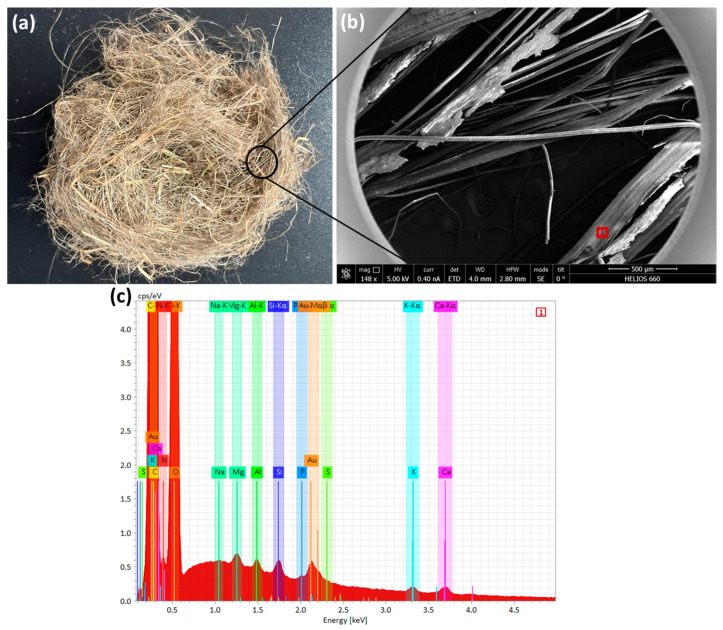
Raw/untreated flax tows used in this study (**a**), SEM image (**b**) and EDS spectrum of fiber surface (**c**).

In this study, fibrous by-products of flax processing were used as soil reinforcement. They were purchased as long fibers and shives in the form of twisted bundles called tows. In a previous study, Martin et al. [20] reported that despite the differences in appearance and morphology of scutched flax and raw flax tows, the tensile properties of single fibers were in the same range. Subsequently, Moothoo et al. [26] and Lazorenko et al. [27,28] confirmed the effectiveness of flax tows for composite applications, allowing FTs to be considered as a sustainable alternative to commercial plant fibers for soil reinforcement at lower cost. The process of preparing the flax tows is illustrated in Figure 2. These flax tows were older than three years. Before using, the FTs were cut with scissors. The fibers have a length of 15 mm and a thickness ranging from 5 to 250 μm (Figure 3). The length of the FTS was determined based on the results of previous studies on the influence of natural plant fibers on soil characteristics [29,30]. The moisture content of the fiber was 12%, the impurity content was 15.5%, and the breaking force of the twisted tape was 155 N. The fiber from the manufacturer displayed a visually uniform distribution with minimal inclusion of conjugated fibers.

### 2.3. Specimen Preparation

Distilled water was added to the pre-dried soil, and the mixture was adjusted to the optimal moisture content before being sifted through a 2 mm sieve. Following this, the flax tows (0.2%, 0.4%, 0.6%, 0.8%, and 1% of the dry weight of the soil) were incorporated into the wet soil. The mixture was then thoroughly stirred until visually homogeneous. It was stored in a hermetically sealed container for 24 h. Subsequently, the mixture was compacted layer by layer into a metal cylinder to form a sample with a diameter of 74 mm and a height of 38 mm, as well as a diameter of 71.4 mm and a height of 20 mm for uniaxial compression and shear tests, respectively. For the desiccation cracking test, distilled water was added to the dry soil mixed with flax tows to achieve a moisture content of 337% (1.2 times above the liquid limit). After homogenization, the resulting paste was placed in small portions into a glass dish (Ø100 mm, h = 13 mm), avoiding visible signs of fiber aggregation and trapped air bubbles by vibrating for 2 min. The resulting samples were then sealed with polyethylene film and cured for 24 h before testing.

### 2.4. Testing Program

The uniaxial compression tests were carried out in accordance with Standard GOST 12248.2 [32]. During these tests, samples were subjected to vertical loading with the ability for unlimited lateral expansion until failure occurred. The loading rate was set at 1 mm/min using the ASIS-1 testing machine (Geotek LLC, Moscow, Russia). The compressive strength $\sigma_{c}$ of the samples was calculated by the following formula:

(1) $$\sigma_{c}=\frac{P}{S^{\prime }}$$

where *P* is the breaking load (N) and *S* is the area of the working surface of the pressure plate (mm^2^).

To assess the impact of flax tows on the shear resistance of expansive soil, shear tests were conducted following Standard 12248.1 [33]. These tests determined the maximum average shear stress at which the soil sample sheared along a fixed plane under normal stress of 0.1 MPa. The shear tests were performed in kinematic mode with a constant loading rate of 1 mm/min using the single-plane shear device SPP-40/35-10 (Geotek LLC, Moscow, Russia). Results from both the compression and shear tests were taken as the arithmetic mean of five parallel measurements to ensure accuracy and reliability.

For the desiccation cracking test, samples were subjected to room conditions with a constant temperature of 25 ± 2 °C and relative humidity of 50 ± 5%. The test was conducted until the samples’ mass loss was complete. Images of crack patterns were obtained using a digital camera positioned 30 cm above the surface of the samples [34]. The obtained images were digitally processed for quantitative analysis of the crack patterns according to the geometric parameters outlined in the procedure described below.

### 2.5. Image Processing and Quantitative Analysis

The photos taken during the experiment were processed using ImageJ software (2024) to quantitatively assess the crack dynamics in FT-reinforced and unreinforced soil. ImageJ, an open-source software based on Java 8, excels in processing grayscale and binary images to extract essential image information such as resolution, color mode, color channels, unit, pixel distribution, and more [35]. The software’s image threshold segmentation function facilitates area measurement and quantitative analysis of porosity as reported by Kravchenko et al. [34]. The following procedure was employed to process the captured images using ImageJ [36]: Grayscale Processing: converting the images to grayscale to simplify further analysis; Binarization: transforming the grayscale images into binary images, where pixels are either black or white, to facilitate crack detection; Denoising: reducing noise in the binary images to improve accuracy during crack analysis.

**Figure 4:**
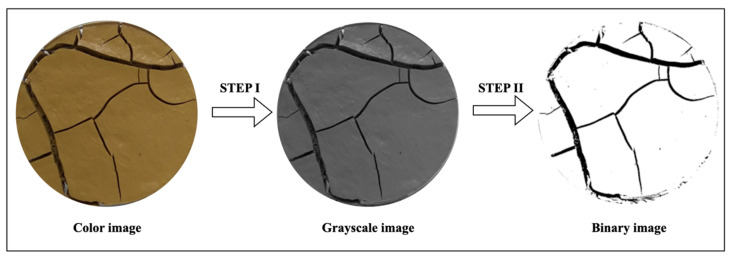
Graphical procedure of measuring the cracks of the clay surface.

The primary parameter for determining the dynamics of crack formation is the crack intensity factor (CIF), which is the ratio of the crack area to the total area in the selected image. Additionally, the crack length density (CLD) was determined, which refers to the length of the crack skeleton per unit area in the selected image [34]. The sequence of photo processing is shown in Figure 4.

## 3. Results and Discussion

### 3.1. Effect of Fiber Content on Soil Shear Strength

**Figure 5:**
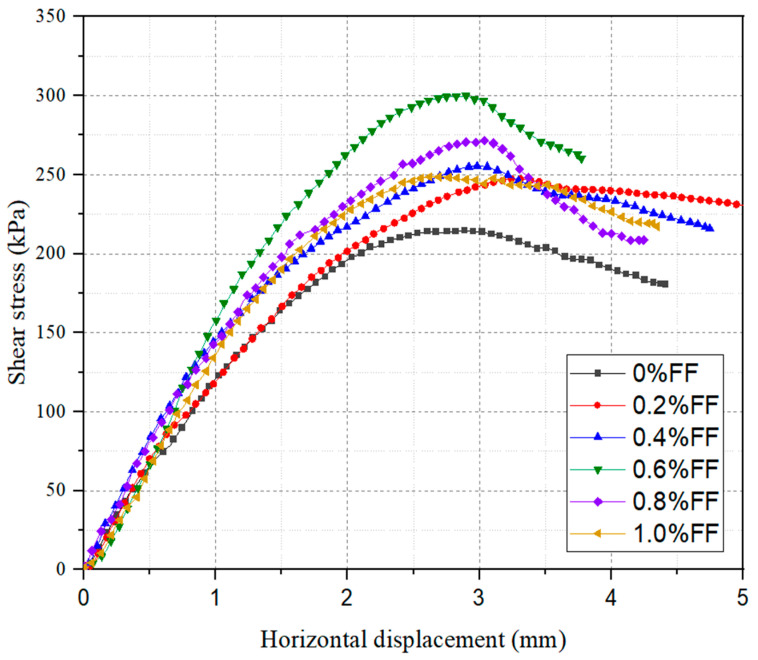
Variation in shear stress with horizontal displacement for FT-reinforced soil.

**Figure 6:**
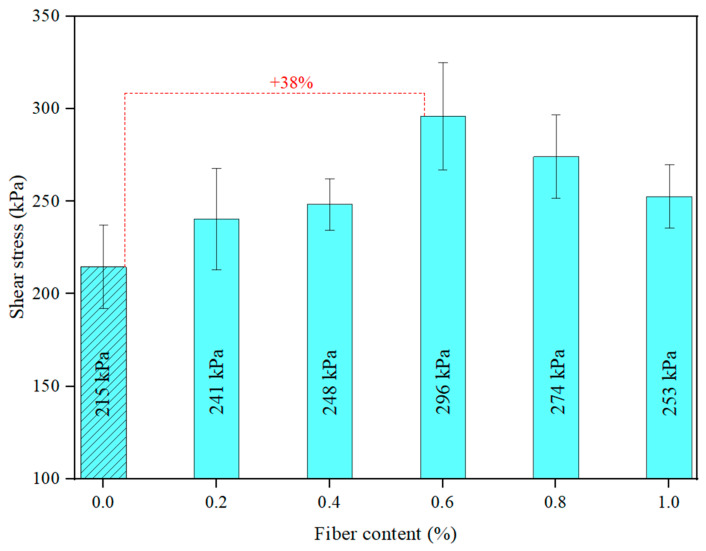
Effect of flax tow content on soil shear strength.

The results of the laboratory tests on shear under different FT contents are shown in Figure 5 and Figure 6. From Figure 5, it can be seen that the unreinforced sample loses shear stress after 2.7% of horizontal displacement. However, with the inclusion of 0.2% flax tows, an increase in horizontal displacement is observed. Furthermore, further increasing the FT content leads to a rise in shear stress and a slight reduction in horizontal displacement. The samples reinforced with flax tows showed a smaller decrease in post-peak strength. This reduction in post-peak shear stress loss becomes more significant with higher vertical normal stresses and greater fiber contents [37]. Consequently, adding flax tow reinforcement seems to increase the residual shear strength angle of the soil. Notably, the most substantial improvement was observed at an FT content of 0.6%, where the shear strength increased to 297 kPa (Figure 6). This represents a 38% improvement over the unreinforced sample, suggesting that relatively modest amounts of FTs can lead to significant enhancements in soil performance. A further increase in the content of randomly distributed discrete flax fibers and shives led to a decline in peak shear strength values after reaching the optimal content. This behavior is explained by the uneven distribution of fibers in the clay, which disrupts the integrity of the soil matrix and leads to a decrease in contact between fibers and clay particles [19]. A similar trend was observed by Wang et al. [38], who used jute fibers to reinforce expansive soil. They explained the strengthening mechanism as the maintenance of soil integrity by natural fibers, which experience tensile stress during shear failure. When the fiber content exceeds the optimal level, the distribution of FTs in the soil matrix becomes more uneven, with some fiber strands locally forming clusters, thereby reducing their contact with soil particles. As a result, the fibrous component of FTs can no longer fully perform its reinforcing function, increasing the tendency for samples to fail under shear stress.

**Figure 7:**
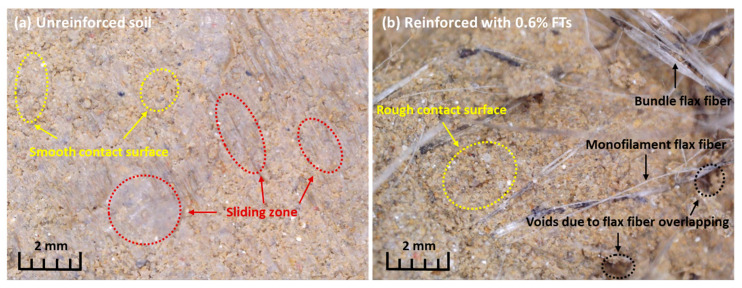
Failure mode of (**a**) unreinforced and (**b**) FT-reinforced expansive soil samples in the simple shear.

The elevated content of low-friction clay mineral particles in the tested expansive soil accounts for the observed sliding shear behavior (Figure 7a). This phenomenon is characteristic of soils with a relatively high clay fraction [39]. The shear failure surface in the unreinforced soil appears relatively smooth (Figure 7a). However, the incorporation of flax tows induces disturbances along the shear surface, leading to an increase in roughness (Figure 7b).

### 3.2. UCS Tests

**Figure 8:**
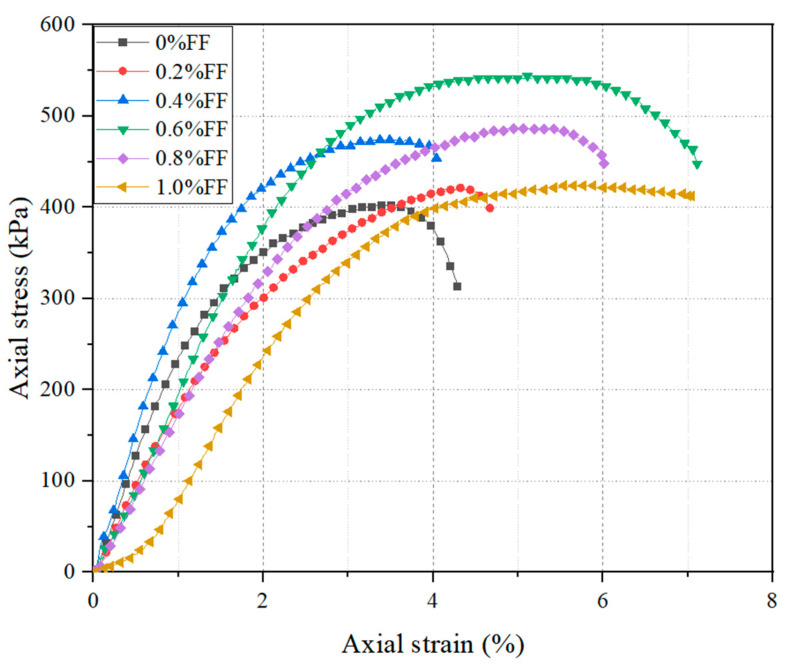
Axial stress–strain behavior of unreinforced and FT-reinforced clay.

The axial stress–strain response of clay containing the different content of flax tows is shown in Figure 8. From this figure, it can be seen that the axial stress of the unreinforced clay reaches 406 kPa, after which the sample fails at 3.5% axial strain. The inclusion of flax fiber at a content of 0.2% increases both the peak axial stress to 432 kPa and the axial strain at failure to 4.4%. The inclusion of 0.4% flax fiber results in a more significant increase in axial stress to 461 kPa, while the axial strain at failure does not increase. The inclusion of 0.6% flax fiber leads to the maximum axial stress–strain ratio at which failure occurs. Further increasing the FT content results in a decrease in this parameter.

During the elastic phase (approximately up to 2% of axial strain), the initial compression of the clay matrix occurs, leading to stress transfer from one grain to another. In reinforced samples, the natural fibers begin to stretch and mobilize their inherent elasticity, contributing a synergistic effect with the expansive clay’s elasticity. This combined effect enhances the overall stiffness and resilience of the composite material. As the deformation progresses into the elasto-plastic phase (approximately after 2% of axial strain), the behavior of the clay-fiber mixture shifts. The FTs, now fully mobilized, significantly increase the elasto-plastic deformation capacity of the composite. This phase is marked by irreversible deformation, where the material does not completely recover its original shape after unloading. The presence of FTs alters the failure mechanism of the clay from brittle to plastic. This transition is due to the flax fibers’ ability to absorb and redistribute stress, delaying the onset of failure and allowing for greater strain accommodation [40].

**Figure 9:**
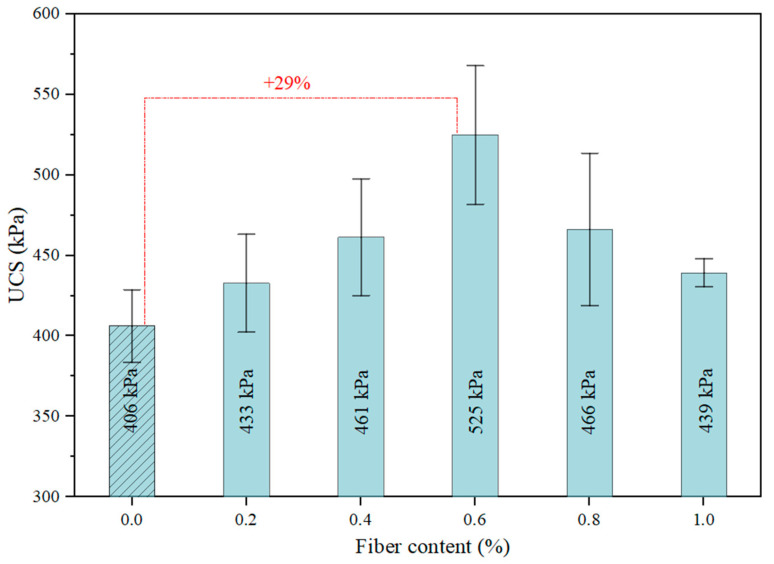
Effect of flax tow reinforcement on the unconfined compressive strength.

The effect of flax fiber inclusion in the soil on its UCS is presented in Figure 9. The results indicate that increasing the flax fiber content up to 0.6% leads to a gradual increase in UCS, with the maximum unconfined compressive strength achieved at a flax tow content of 0.6%. This FT content results in a 29% increase in UCS compared to unreinforced soil, reaching 522 kPa. Further increasing the flax tow content leads to the opposite effect—a decrease in UCS. At low FT content, the distance between fibers within the soil matrix is significant, resulting in less efficient formation of the fiber–soil network. As the flax tow content gradually increased, the distance between the fibers decreased, promoting the formation of a stronger three-dimensional reinforcing network within the soil matrix. This enhanced the interaction between soil particles and allowed for more uniform load distribution during compression. This effect was most pronounced at an FT content of 0.6%. Incorporating higher percentages of flax tows leads to fiber entanglement, reducing the sample’s uniformity, thereby increasing the likelihood of segregation between the soil particles and flax fibers, as well as the formation of more clumps and voids. This may explain the decrease in peak compressive strength of the reinforced soil when the FT content exceeds 0.6%.

The increase in the strength of expansive clay by 29% with the application of just 0.6% fiber facilitates the use of expansive clays for the construction of various geotechnical structures. The incorporation of fiber enhances the engineering properties of the soil while maintaining economic efficiency.

**Figure 10:**
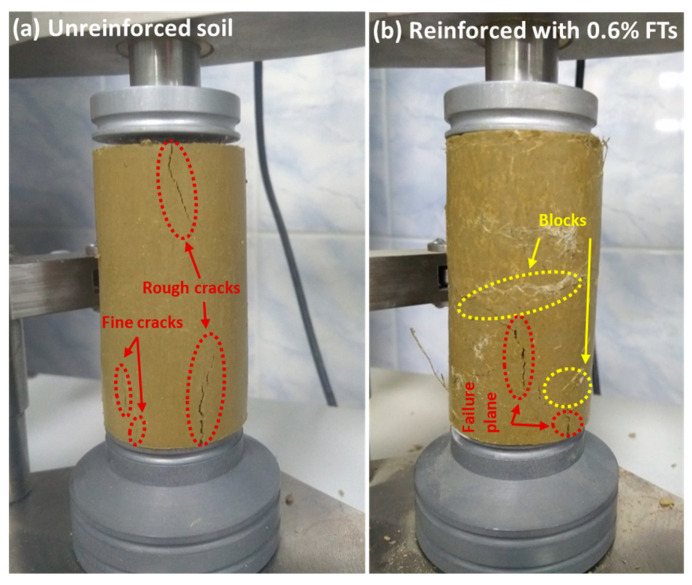
Failure mode of (**a**) unreinforced and (**b**) FT-reinforced expansive soil samples in UCS test.

It was found that the inclusion of FTs reduces sample deformation at failure and mitigates its brittle failure behavior (Figure 10). When the samples are subjected to compressive forces, the FTs act as tensile elements, providing a restraining effect and effectively limiting the lateral and radial deformation of the soil. Randomly distributed discrete flax fibers and shives also contributed to containing the cracks forming within the soil matrix, preventing further failure (Figure 10b).

### 3.3. Crack Parameters

**Figure 11:**
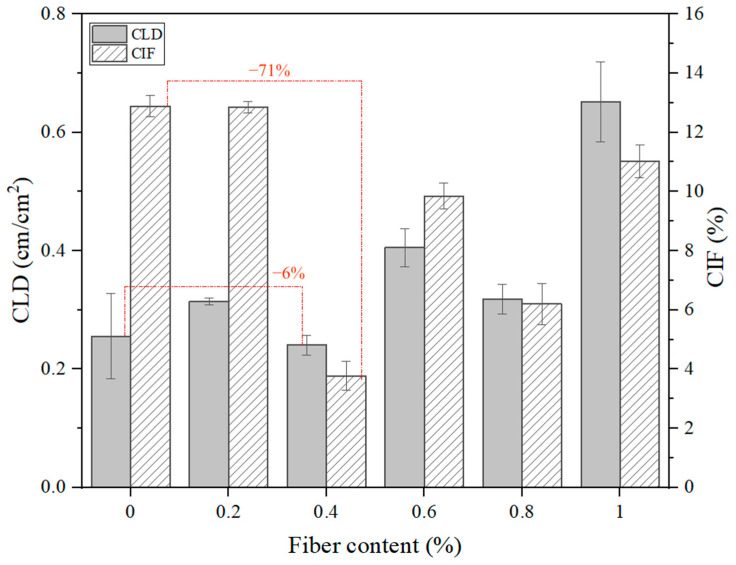
The impact of flax tow inclusion on clay crack parameters.

Crack formation parameters such as CIF and CLD are presented in Figure 11. The unreinforced soil, after complete drying, had distinct contours of widely opened cracks, forming well-defined soil plates. The total crack length of unreinforced clay initially was 43.9 cm, which corresponds to a CLD of 0.25 cm/cm^2^. This is mainly because of the high montmorillonite content of clay, which attracts and retains water within the crystalline double layers of molecules [41]. When desiccation occurs, water is removed from these layers, leading to overall volumetric reduction and crack formation. With the inclusion of 0.2% flax tows, this indicator slightly increased, showing an increase in the total crack length. With the inclusion of 0.4% flax tows, the smallest CLD value was observed, indicating a decrease of 6% compared to the unreinforced soil sample. Further increasing the FT content led to a noticeable increase in crack lengths as well as fine cracking. Small cracks without significant openings were observed on the soil surface, and with the addition of 1% FTs, the maximum CLD value of 0.64 cm/cm^2^ was reached.

The CIF results for unreinforced and 0.2% FT-reinforced clay showed similar outcomes, amounting to 13%. With 0.4% flax tow content, this parameter decreased by 71% compared to the unreinforced sample. Such a significant reduction is due to a substantial decrease in crack opening area as well as crack length, which affected the final CIF. However, with further increases in flax tow content, an increase in CIF was observed, primarily due to the increase in crack lengths rather than crack openings.

**Figure 12:**
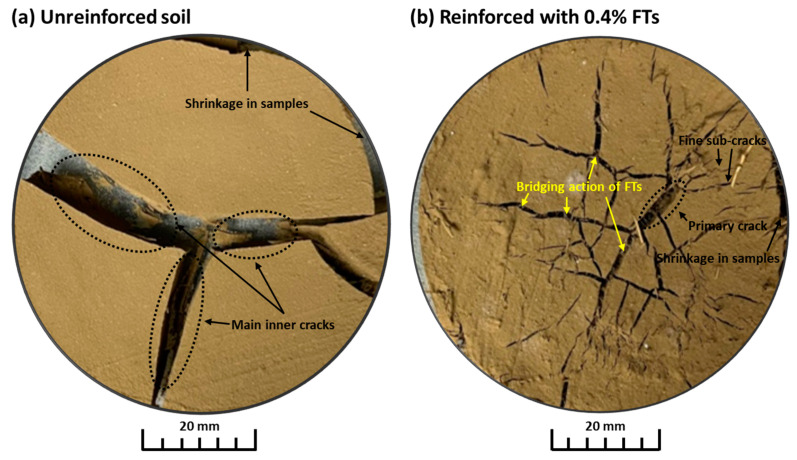
Final crack patterns of (**a**) unreinforced and (**b**) FT-reinforced expansive soil samples in desiccation tests.

This study demonstrates that adding flax by-products to expansive soil reduces soil cracking. Initially, the sample contains flax tows, soil, water, and air, with fibers randomly distributed throughout the soil. The reinforced samples exhibited non-orthogonality and an irregular network of cracking (Figure 12).

This was also observed in the work of [41], and a possible explanation could be the uneven drying of the sample when fiber is included. It is known that flax fibers are hollow and can absorb water up to 20% of their own weight [42], which could contribute to better moisture regulation within the soil matrix. This property allows the fiber to facilitate water migration within the soil, thereby helping to mitigate tensile stress and reduce the likelihood of soil cracking. As water evaporates, the sample shrinks both horizontally and vertically. As moisture decreases, cracks gradually propagate from the sample’s surface to the fibers, which are embedded on both sides of the crack. At this stage, the fiber and soil act as a “skeleton structure”, effectively limiting soil cracking due to the friction between the fibers and the soil; this is also confirmed in the work of [43]. The fibers provide tensile resistance, reducing further crack widening. When water movement is obstructed by fibers, the migration of water is impeded, increasing the length of the water flow path and reducing the cross-sectional area available for water flow.

Flax tows are randomly distributed in the clay, as shown in Figure 11. When flax fibers are mixed with clay, they bind with clay particles, thereby strengthening the inter-particle bonds, as confirmed by [19]. Additionally, the integrity of the reinforced clay is increased, the deformation and displacement of the clay particles are effectively restricted, and the cohesion of the reinforced clay is enhanced. Consequently, the shear strength of the reinforced clay is improved. An optimal flax tow content of 0.4% in expansive soils helps reduce crack formation, which positively impacts the strength characteristics. This effect is beneficial for the practical geoengineering applications of natural fibers. It should be noted that the cracking resistance and mechanical performances of these materials are closely related to the quality of the interface between the soil matrix and the flax fibers [44,45,46]. Thus, the search for effective methods of surface modification of FTs to improve the fiber–soil matrix interface is of interest for further studies.

## 4. Conclusions

In this study, laboratory investigation was conducted to investigate the properties of flax tows-reinforced expansive soil. Laboratory tests on shear, uniaxial compression, and crack formation parameters of clayey soil reinforced with various contents of randomly distributed flax tows were performed. The main conclusions of this study are presented below:

(1) The results of the UCS test showed an increase in the maximum axial compressive stress of FT-reinforced expansive soil by up to 29%. The optimal dosage of FTs was 0.6%, which led to the entanglement and agglomeration of the flax fibers. This issue reduced the ability of the flax fibers to effectively interact with the soil matrix, thereby lowering the peak stress. A moderate content of flax tows altered the failure behavior of the swelling soil from brittle to more plastic.
(2) The change in shear strength of the reinforced soil demonstrated a similar trend to the UCS results. The maximum post-peak strength was achieved with the inclusion of 0.6% flax tows, which exceeded the shear strength of the unreinforced soil by 38%. A further increase in FT content led to an uneven distribution of fibers within the soil matrix, fiber overlapping, and a reduction in strength due to lower frictional resistance between fibers compared to the fiber–soil interaction.
(3) Soil desiccation cracks were greatly decreased when an FT reinforcement was used. With an increasing fiber content, the crack index factor decreased by 71%, with 0.4% FT dosage, and the crack length density decreased by 6% in the soil reinforced with 0.4% flax tows, as compared to the unreinforced clay soil. This was mostly due to the randomly distributed discrete flax fibers and shives inclusion, which boosted soil cracking resistance substantially.

Overall, the experimental results demonstrated that using flax tows, a highly renewable and environmentally friendly by-product of flax production, can significantly improve the mechanical and cracking characteristics of soils with a high content of swelling clay minerals. Therefore, chopped-short FTs can serve as a suitable candidate for reinforcing expansive soils. The search for effective methods of surface modification of FTs to improve the adhesion between fiber and the soil matrix is of interest for further studies. The mixing mechanisms and mixing processes should also be optimized to improve the homogeneity of the distribution of FTs in the soil matrix.

## Figures and Tables

**Table 1:** Composition and physical properties of soil used in this study.

Soil Properties	Testing Method	Value
Chemical composition (wt%)	XRF analysis	
SiO_2_		69.1
Al_2_O_3_		22.7
Fe_2_O_3_		6.5
TiO_2_		0.8
K_2_O		0.3
CaO		0.1
Mineral composition (wt%)	XRD analysis	
Montmorillonite		69.5
Quartz		24.5
Kaolinite		2.7
Calcite		1.2
Albite		2.0
Atterberg limits	ASTM D 4318-17 (2017) [21]	
Liquid limit (%)		280.9
Plastic limit (%)		32.3
Plasticity index (%)		248.6
Normal proctor characteristics	ASTM D 698-07 (2007) [22]	
Maximum dry density (g/cm^3^)		1.72
Optimum moisture content (%)		39.5
Particle characteristics	Laser Diffraction Particle Size Analysis	
D10 (μm)		24.1
D50 (μm)		52.2
D90 (μm)		87.9
Other properties		
Specific gravity	ASTM D 854 (2010) [23]	2.7
Axial swelling strain (%)	GOST 12248.6 (2020) [24]	27.1
USCS classification	ASTM D 2487 (2011) [25]	CH

## Data Availability

The raw data supporting the conclusions of this article will be made available by the authors on request.
